# Elevating Supercapacitor Performance of Co_3_O_4_-g-C_3_N_4_ Nanocomposites Fabricated via the Hydrothermal Method

**DOI:** 10.3390/mi15030414

**Published:** 2024-03-20

**Authors:** Manesh A. Yewale, Vineet Kumar, Aviraj M. Teli, Sonali A. Beknalkar, Umesh T. Nakate, Dong-Kil Shin

**Affiliations:** 1 School of Mechanical Engineering, Yeungnam University, Gyeongsan 38541, Republic of Korea; maneshphd@gmail.com (M.A.Y.); vineetfri@gmail.com (V.K.); 2 Division of Electronics and Electrical Engineering, Dongguk University-Seoul, Seoul 04620, Republic of Korea; avteli.teli@gmail.com (A.M.T.); sonaliabeknalkar@gmail.com (S.A.B.); 3 Department of Polymer-Nano Science and Technology, Jeonbuk National University, 567 Baekje-daero, Deokjin-gu, Jeonju-si 54896, Republic of Korea

**Keywords:** graphic carbon nitride (g-C_3_N_4_), Co_3_O_4_ nanoparticles, supercapacitor

## Abstract

The hydrothermal method has been utilized to synthesize graphitic carbon nitride (g-C_3_N_4_) polymers and cobalt oxide composites effectively. The weight percentage of g-C_3_N_4_ nanoparticles influenced the electrochemical performance of the Co_3_O_4_-g-C_3_N_4_ composite. In an aqueous electrolyte, the Co_3_O_4_-g-C_3_N_4_ composite electrode, produced with 150 mg of g-C_3_N_4_ nanoparticles, revealed remarkable electrochemical performance. With an increase in the weight percentage of g-C_3_N_4_ nanoparticles, the capacitive contribution of the Co_3_O_4_-g-C_3_N_4_ composite electrode increased. The Co_3_O_4_-g-C_3_N_4_-150 mg composite electrode shows a specific capacitance of 198 F/g. The optimized electrode, activated carbon, and polyvinyl alcohol gel with potassium hydroxide were used to develop an asymmetric supercapacitor. At a current density of 5 mA/cm^2^, the asymmetric supercapacitor demonstrated exceptional energy storage capacity with remarkable energy density and power density. The device retained great capacity over 6k galvanostatic charge–discharge (GCD) cycles, with no rise in series resistance following cyclic stability. The columbic efficiency of the asymmetric supercapacitor was likewise high.

## 1. Introduction

A crucial and fundamental challenge to energy storage systems is the use of pollution-free and long-life energy technology for storage. The requirement for renewable energy sources and energy storage devices has grown dramatically in the recent decade as the usage of nonrenewable energy sources has increased, resulting in increased pollution [1,2]. To fulfill demand while reducing pollution, innovative research on energy sources and energy storage systems is essential. Batteries are alternative forms of energy storage that are utilized to store direct renewable energy from renewable sources; however, their power density is constrained. Because of their unique properties, such as quick charging times, longevity, excellent power density, and cost-effectiveness in manufacturing, supercapacitors are an attractive alternative energy storage solution for renewable energy. They are also thought to be safer than batteries [3,4]. Supercapacitors are classified into two classes based on charge storage mechanism: Electric double-layer supercapacitors (EDLC) store charge by ion adsorption, with most carbon-based materials exhibiting the EDLC storage mechanism except for select metal oxides such as RuO_2_. The other type of capacitor is a pseudocapacitor, which stores charge via faradic processes. Metal oxides and chalcogenides, such as NiCo_2_O_4_, are examples of materials that store charge via the pseudocapacitor mechanism. The electrochemical performance of supercapacitors must be studied in order to determine their efficiency [5]. Several metal oxides, metal chalcogenides, conducting polymers, and metal hydroxides have been explored for supercapacitor applications, as have composites with carbon-based materials like carbon nanotubes and graphene oxide. Transition metal oxides have attracted significant attention owing to their unique characteristics, such as chemical stability and variable valence [6]. As a result of its superior electrochemical performance, reversibility, and larger theoretical capacity of 3560 F/g [7,8], the transition metal oxide Co_3_O_4_ has gained increased recognition as a competent electrode material for supercapacitor applications [8]. Several approaches to synthesizing nanoparticles of Co_3_O_4_ for use in energy storage have been reported in the recent decade, including electrodeposition, chemical bath deposition, co-precipitation, spray pyrolysis, sol–gel, electrospinning, and others. Surface morphologies of Co_3_O_4_ nanoparticles, including flowers and nanorods, have been observed, indicating surface-dependent supercapacitor performance. Due to its unique properties, a composite with a carbon-based material like g-C_3_N_4_ is an ideal candidate for improving the supercapacitor performance and energy density of the electrode material. The presence of pyrrolic nitrogen-hole defects inside the lattice, as well as the increased distance of nitrogen atoms covalently bonded at the edges, increases the material’s rate capacity. Furthermore, nitrogen sp^2^ hybridization affords coordination sites, and the porous structure of heptazine contributes to these benefits [9,10,11,12,13,14,15,16]. Graphitic carbon nitride (g-C_3_N_4_) is an arrangement with a lamellar structure that is inherently nitrogen-rich. Apart from being the most stable allotrope of carbon nitrides in the environmental atmosphere, it offers rich surface characteristics that make it suitable for a variety of applications, such as hydrogen evolution and supercapacitor electrodes. The weak van der Waals interaction between the layers provides the 2D lamellar structure, leading to the polymeric structure of g-C_3_N_4_. Nitrogen’s lone pair of electrons may give the electrode material surface polarity, which might offer a number of binding sites for the electrolyte ions to participate in surface interaction [5,17]. Inserting Co_3_O_4_ nanoparticles into g-C_3_N_4_ nanoparticles creates an active surface, which improves surface polarity, electrolytic performance, wettability, and electrical conductivity. This alteration will have an effect on the electrochemical performance and energy storage capabilities of Co_3_O_4_ nanoparticles [18,19,20,21,22]. We propose a hydrothermal approach for making Co_3_O_4_-g-C_3_N_4_ composites, while investigating the material’s electrochemical performance. We synthesized Co_3_O_4_-g-C_3_N_4_ composites that had various g-C_3_N_4_ nanoparticle weight percentages. Using 150 mg of g-C_3_N_4_ nanoparticles, the electrode and device demonstrated beneficial electrochemical performance. The composite of Co_3_O_4_ and 150 mg of g-C_3_N_4_ enhanced the electrical conductivity of the Co_3_O_4_-g-C_3_N_4_ nanocomposite, and the effects were observed in the electrochemical performance of the electrode and the ASC. The improved electrode’s practical use in aqueous electrolytes yields impressive results. Two-electrode experiments show that the Co_3_O_4_-g-C_3_N_4_ composites are an acceptable choice for supercapacitor applications.

## 2. Materials and Methods

### 2.1. Preparation of Graphitic Carbon Nitride (g-C_3_N_4_)

A simple thermal technique was used for making graphitic carbon nitride (g-C_3_N_4_). Melamine (5 g) was put in an alumina crucible and heated at a rate of 5 degrees per minute for 4 h, achieving a temperature of 550 degrees. The resultant product was observed as yellow-colored graphitic carbon nitride (g-C_3_N_4_) when the reaction was completed.

### 2.2. Preparation of Cobalt Oxide-Graphitic Carbon Nitride (Co_3_O_4_-g-C_3_N_4_)

**Scheme 1:**
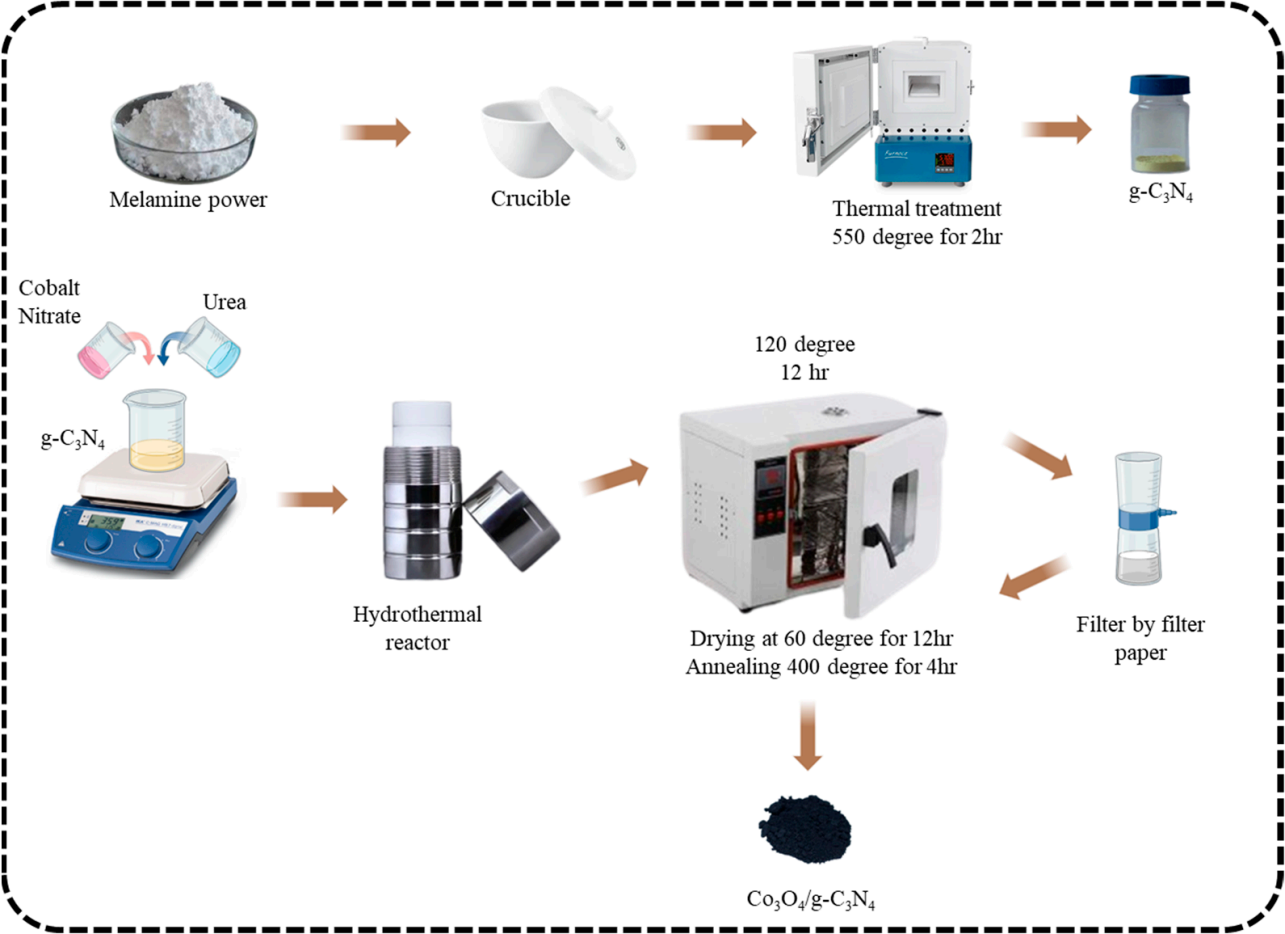
Schematic representation for synthesis of g-C_3_N_4_ and hydrothermal synthesis of Co_2_O_4_-g-C_3_N_4_ composite.

The Co_3_O_4_-g-C_3_N_4_ nanocomposite material was synthesized using a hydrothermal method. To begin, 100 mM cobalt nitrate was dissolved in 40 mL of deionized water and stirred for 10 min to form a homogenous solution. After that, 400 mM urea was added to the aforesaid solution and stirred for another 10 min. Finally, 50 mg of g-C_3_N_4_ nanoparticles was added and mixed for 10 min more. After that, the solution was put into a 100 mL Teflon liner for the hydrothermal process. The hydrothermal reactor was hermetically sealed and kept at 120 degrees Celsius for 12 h. The reaction mixture naturally cooled when the reaction was complete. The product obtained was washed multiple times with ethanol and water before being filtered through filter paper. The filtered product was oven-dried overnight at 60 degrees. In a muffle furnace, the dry nanoparticles were annealed at 400 degrees for 4 h. The resultant compound was designated as Co_3_O_4_-g-C_3_N_4_-50 mg, and the same nomenclature was utilized for subsequent analysis. A similar procedure was employed to make the Co_3_O_4_-g-C_3_N_4_ composites with weight percentages of 100 mg, 150 mg, 200 mg, and 250 mg which were named Co_3_O_4_-g-C_3_N_4_-100 mg, Co_3_O_4_-g-C_3_N_4_-150 mg, Co_3_O_4_-g-C_3_N_4_-200 mg, and Co_3_O_4_-g-C_3_N_4_-250 mg respectively. The schematic representation of the synthesis of g-C_3_N_4_ and the synthesis of Co_3_O_4_-g-C_3_N_4_ composites is shown in Scheme 1.

### 2.3. Preparation of Electrode

A three-electrode arrangement was used for the electrochemical examination of the developed Co_3_O_4_-g-C_3_N_4_ composites. The composite material was used to make the working electrode; platinum served as the counter electrode; and an Ag/AgCl electrode functioned as the reference electrode. The working electrode was prepared by slurrying the active electrode material, carbon black, and PVDF in an NMP solution with an 80:10:10 weight percentage. The resulting set was then drop-cast onto 1 × 1 cm^2^ of nickel foam. Before electrode preparation, the nickel foam was thoroughly cleaned with water, ethanol, and acetone, each with 10 min of ultra-sonication. Electrochemical experiments were carried out in a 2 M KOH aqueous electrolyte. The two-electrode arrangement was performed using an optimized electrode as the working electrode, while an activated carbon electrode was used as the counter electrode. The activated carbon electrode is prepared in the same way as the working electrode, with the sole variation being that activated carbon is used instead of active material. To create the two-electrode arrangement, polyvinyl alcohol (PVA) and KOH gel were used as electrolytes. The gel was prepared using the same approach as described in a previous article. 

### 2.4. Equations

The b value, diffusion coefficient, transfer coefficient, standered rate constant, capacitice and diffusion contribution, specific capacitance, energy density and power density of the all electrode were calculated from following Equations (1)–(7) below [23]

(1) $$i=a\times v^{b}=\log\left(i\right)=b\cdot \log\left(v\right)+log(a)$$

(2) ip=0.4463×A×F×C×nFDvRT

(3) ip=0.227 AFCnk0−nFαRTEp−E0

(4) $$i\left(V\right)=k_{c}v+{k_{d}v}^{0.5};\;\frac{i\left(V\right)}{v^{0.5}}={k_{c}/v}^{0.5}+k_{d}$$

(5) $$\mathrm{Specific}\;\mathrm{Capacitance}\;(\mathrm{Cs})=\frac{T_{d}\times I_{d}}{dV\times m}$$

(6) $$\mathrm{Energy}\;\mathrm{Density}\;(\mathrm{EDs})=\frac{C_{s}\times {dV}^{2}}{7200}$$

(7) $$\mathrm{Power}\;\mathrm{Density}\;(\mathrm{PDs})=\frac{{ED}_{s}\times 3600}{T_{d}}$$

## 3. Results and Discussion

### 3.1. XRD Analysis

**Figure 1:**
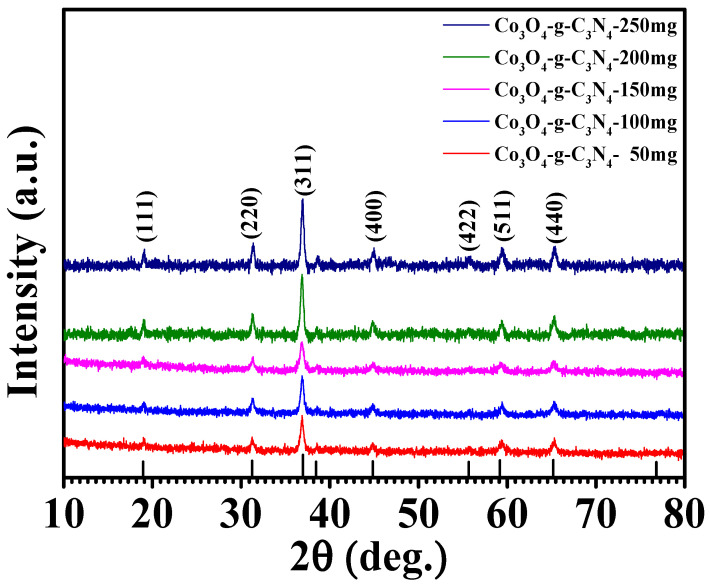
XRD spectra of Co_3_O_4_-g-C_3_N_4_ composite for different weight percentages of g-C_3_N_4_ nanoparticles.

The phase structure and structure of the crystals of the developed nanoparticles have been determined by XRD analysis. The XRD spectra of the Co_3_O_4_ nanoparticle composite with various weight percentages of graphitic carbon nitride, which vary from 50 to 250 mg, are shown in Figure 1. The phases apparent in the Co_3_O_4_ nanoparticles are shown in the XRD spectra. XRD spectra from all compositions correspond well with the JCPDS card number 00-001-1152, with (hkl) planes (111), (220), (311), (400), (422), (511), and (440) at glancing angles of 18.95°, 31.30°, 36.82°, 44.97°, 55.86°, 59.35°, and 65.22°. The cubic crystal structure of the Co_3_O_4_ nanoparticles is confirmed by the (hkl) plane matching with the standard data. The development of nanoparticles is seen at an angle of 36.82° along the (311) crystal planes. The intensity of the XRD spectra changes with the g-C_3_N_4_ content. The peak strength of the (311) and (440) peaks diminishes when g-C_3_N_4_ in the Co_3_O_4_-g-C_3_N_4_ composition increases, perhaps indicating the creation of the Co_3_O_4_-g-C_3_N_4_ composite.

### 3.2. XPS Analysis

**Figure 2:**
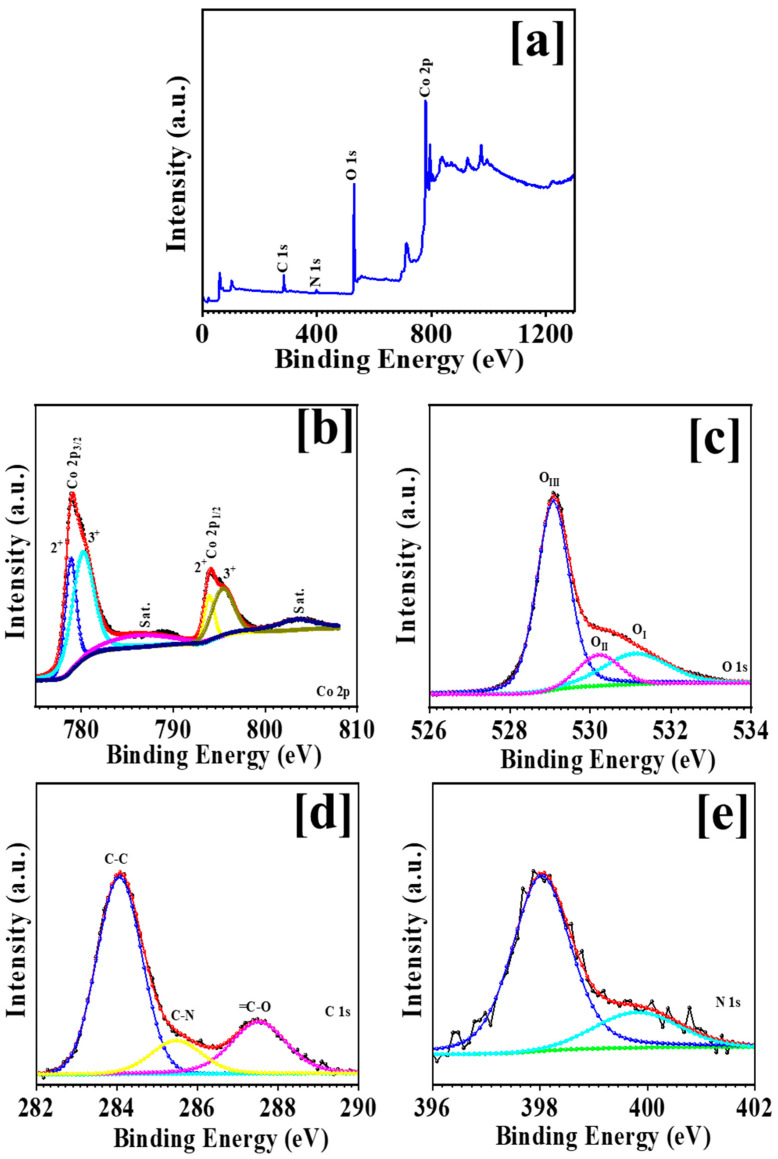
XPS spectra (**a**) survey scan, (**b**) Co 2p, (**c**) O 1s, (**d**) C 1s, and (**e**) N 1s of Co_3_O_4_-g-C_3_N_4_-150 mg nanoparticles.

X-ray photoelectron spectroscopy (XPS) was implemented to examine the chemical state and elemental composition of the improved nanoparticle. The survey scan spectra of the Co_3_O_4_-g-C_3_N_4_-150 mg nanoparticles are shown in Figure 2a, revealing prominent peaks for cobalt, oxygen, carbon, and nitrogen observed at 779.21 eV, 529.1 eV, 284.17 eV, and 398.02 eV, respectively, and the occurrence of all elements in the optimized electrode.

Figure 2b shows the convolution and deconvolution Co 2p spectra, with peaks appearing at 778.98 eV and 794.07 eV, respectively, confirming the existence of Co 2p_3/2_ and Co 2p_1/2_ states. Similarly, two peaks at 779.03 eV and 793.91 eV indicate the presence of the Co^2+^ state, while another two peaks at 780.26 eV and 795.47 eV confirm the presence of the Co^3+^ state. Furthermore, two satellite peaks at 788.72 eV and 803.61 eV are characteristic of Co^2+^ [23,24,25]. The oxygen deconvolution spectra are displayed in Figure 2c, with three peaks at 529.07 eV, 530.24 eV, and 531.21 eV. The presence of metal oxide (Co-O) is demonstrated by the peak at 529.07 eV, whereas the presence of oxygen ions in nanoparticles and adsorbed water is shown by the peaks at 530.24 eV and 531.21 eV, respectively [26,27,28]. Figure 2d additionally displays the carbon deconvolution spectra, which have three separate peak locations at 287.53 eV, 285.51 eV, and 284.10 eV, representing carbon bonds in C=O, C-N, and C-C, respectively [29,30,31]. Figure 2e depicts the deconvolution spectra of the N 1s spectra, with two peaks at 399.85 eV and 398.04 eV corresponding to the C-C and C=C-N bonds, respectively. The XPS study shows the elemental composition of the Co_3_O_4_-g-C_3_N_4_-150 mg nanocomposite is 21.03%, 3.52%, 48.56%, and 26.89% of C, N, O, and Co, respectively, and the XPS results agree with the EDS data, indicating that the Co_3_O_4_-g-C_3_N_4_-150 mg nanoparticle composite phase was formed.

### 3.3. FESEM Analysis

**Figure 3:**
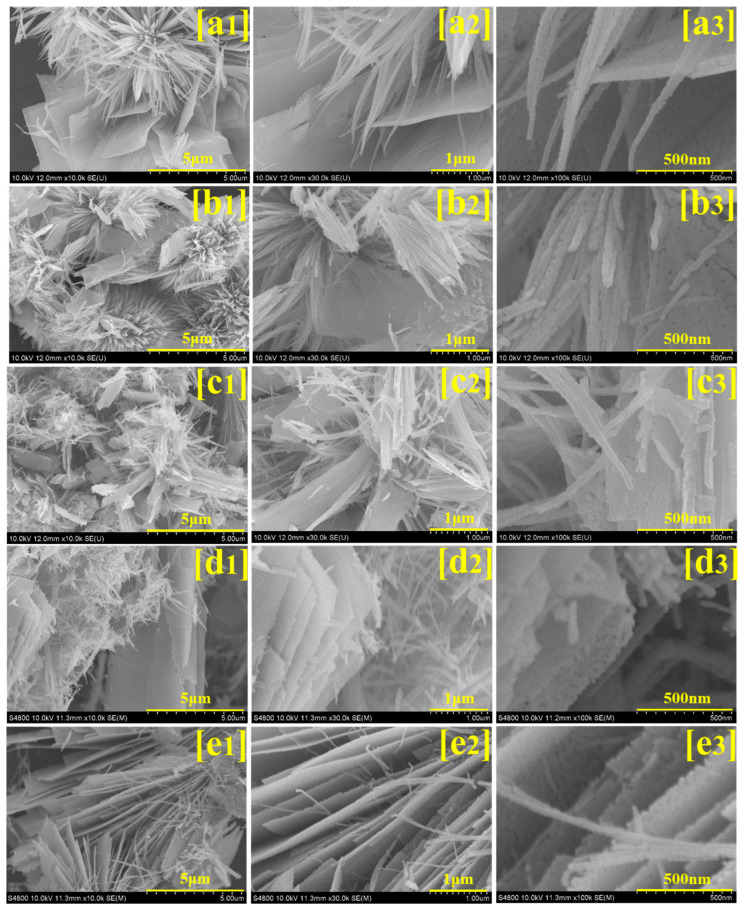
FESEM micrograph of Co_3_O_4_-g-C_3_N_4_ composite for different weight percentages of g-C_3_N_4_ nanoparticles at different magnifications: (**a1**–**a3**) Co_3_O_4_-g-C_3_N_4_-50 mg, (**b1**–**b3**) Co_3_O_4_-g-C_3_N_4_-100 mg, (**c1**–**c3**) Co_3_O_4_-g-C_3_N_4_-150 mg, (**d1**–**d3**) Co_3_O_4_-g-C_3_N_4_-200 mg, (**e1**–**e3**) Co_3_O_4_-g-C_3_N_4_-250 mg.

Field electron scanning microscopy (FESEM) was implemented to examine the surface morphology of the Co_3_O_4_-g-C_3_N_4_ nanocomposite. Figure 3 illustrates a FESEM micrograph of a Co_3_O_4_-g-C_3_N_4_ nanocomposite with various magnifications and g-C_3_N_4_ weight percentages. Figure 3(a1–a3) shows an FESEM image of a Co_3_O_4_-g-C_3_N_4_-50 mg nanocomposite. Co_3_O_4_ microflowers are connected to the g-C_3_N_4_ surface, and Co_3_O_4_ flowers distribute all over the g-C_3_N_4_ layers. The g-C_3_N_4_ layers offer an extensive surface area for electrolyte interaction as well as electrical pathways for charge transfer. The layered structure of g-C_3_N_4_ may be the primary reason for the Co_3_O_4_-g-C_3_N_4_ nanocomposite’s improved electrochemical performance. The impact of raising the weight percentage on the surface microstructure of the Co_3_O_4_-g-C_3_N_4_ nanocomposite is apparent. The g-C_3_N_4_ sheets begin to insert at the core of the Co_3_O_4_ flower for Co_3_O_4_-g-C_3_N_4_-100 mg, as shown in Figure 3(b1–b3), extending the electrolytic interaction route. The flower structure begins to shatter as a result of the insertion of g-C_3_N_4_ sheets, and floral wires become loose and sprinkled on the g-C_3_N_4_ layers, therefore expanding the area of the electrode material. This effect may also be seen in XRD, where the peak intensity diminishes as the g-C_3_N_4_ % increases. Its influence may also be seen in the cyclic voltammetry (CV) and galvanostatic charge–discharge (GCD) profiles. By shattering the flower-like morphology by the insertion of g-C_3_N_4_ sheets, the Co_3_O_4_ blooms are fully disrupted into a wire-like structure for Co_3_O_4_-g-C_3_N_4_-150 mg, as shown in Figure 3(c1–c3). These wires are dispersed throughout the surface and function similarly to wires for charge conductivity in supercapacitors via the g-C_3_N_4_ sheets.

**Figure 4:**
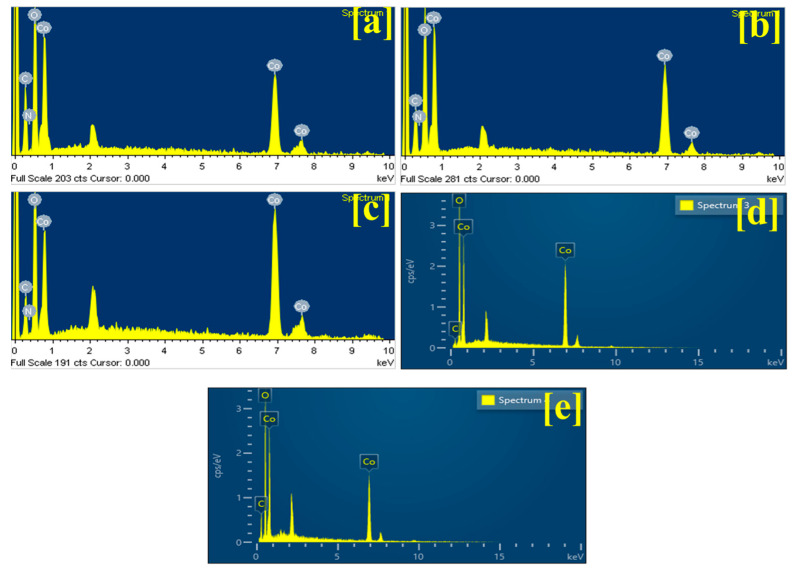
EDS spectra of Co_3_O_4_-g-C_3_N_4_ composite for different weight percentages of g-C_3_N_4_ nanoparticles: (**a**) Co_3_O_4_-g-C_3_N_4_-50 mg, (**b**) Co_3_O_4_-g-C_3_N_4_-100 mg, (**c**) Co_3_O_4_-g-C_3_N_4_-150 mg, (**d**) Co_3_O_4_-g-C_3_N_4_-200 mg, (**e**) Co_3_O_4_-g-C_3_N_4_-250 mg.

The surface morphology of the Co_3_O_4_-g-C_3_N_4_ nanocomposite underwent a dramatic change with further increases in the weight percentage of g-C_3_N_4_, from 200 mg to 250 mg. In the FESEM images for Co_3_O_4_-g-C_3_N_4_-200 mg, a small number of flower wires were observed on the g-C_3_N_4_ nanosheet, as depicted in Figure 3(d1–d3). The effect of this change was observed in the electrochemical performance of the electrode. Similarly, for Co_3_O_4_-g-C_3_N_4_-250 mg, an almost continuous g-C_3_N_4_ sheet was observed, as shown in Figure 3(e1–e3). All Co_3_O_4_-g-C_3_N_4_ nanocomposites were elementally studied through energy-dispersive X-ray spectroscopy (EDS) spectrum measurements. The EDS spectra of the Co_3_O_4_-g-C_3_N_4_ nanocomposite are shown in Figure 4a–e. The EDS spectra of all nanocomposites show distinct peaks for Co, N, C, and O, revealing the formation of the Co_3_O_4_-gC_3_N_4_ composite. The peak intensity and atomic percentage of each element were tuned with changes in weight percentage of g-C_3_N_4_, and the impact was observed in the electrochemical performance of the electrode. The Co_3_O_4_-g-C_3_N_4_-150 mg electrode attained stoichiometry, and the effect was observed by it showing remarkable electrochemical performance compared to the other electrodes.

### 3.4. Transmission Electron Microscopy (TEM) Analysis

**Figure 5:**
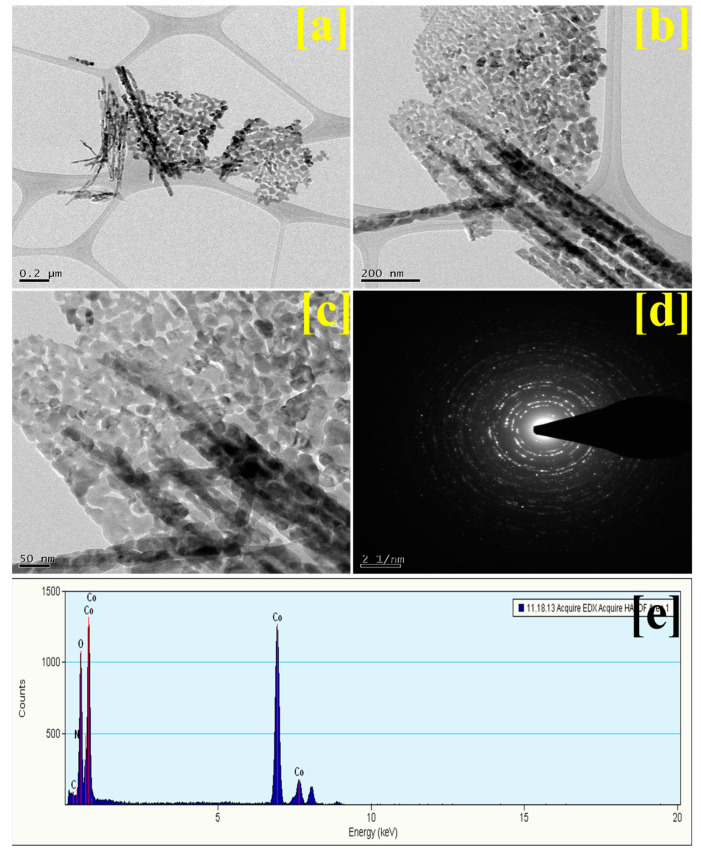
(**a**–**c**) TEM micrographs at differing magnification; (**d**) SEAD pattern; (**e**) EDS spectra of Co_3_O_4_-g-C_3_N_4_-150 mg nanoparticles.

Transmission electron microscopy (TEM) was employed to examine the precise microstructure of the Co_3_O_4_-g-C_3_N_4_-150 mg nanocomposite’s nanoparticles. Figure 5a,b displays TEM micrographs of the Co_3_O_4_-g-C_3_N_4_-150 mg nanocomposite’s nanoparticles under various magnifications. The illustration shows that Co_3_O_4_ nanowires are evenly distributed over the g-C_3_N_4_ nanosheets. Each nanowire makes full contact with the sheet, acting as an electrical network for the flow of charge all through the supercapacitor’s charging and discharging processes.

Figure 5c also shows a higher magnification of the Co_3_O_4_-g-C_3_N_4_-150 mg nanocomposite’s TEM image, illustrating clearly that the g-C_3_N_4_ nanosheets and Co_3_O_4_ nanowires are made up of tiny, nonspherical-shaped nanoparticles. They are arranged horizontally to generate a two-dimensional sheet-like structure and vertically to form nanowire-like structures. Both tiny wires and sheets contain empty spaces that offer accessible spots for the electrolyte, overcome tiny impediments, and increase the surface area for electrode and electrolyte interaction, hence, boosting the Co_3_O_4_-g-C_3_N_4_-150 mg nanocomposite electrode’s energy storage capacity. Figure 5d shows the SAED pattern of the Co_3_O_4_-g-C_3_N_4_-150 mg nanocomposite’s composition, indicating its polycrystalline nature and confirming a good agreement with the XRD spectrum. The lattice parameters hkl plan (400) of the XRD were well matched with the SAED pattern represented in Figure 5d by the yellow ring. Similar results were reported by M. G. Kim et al. [32] and M. Xlao et al. [33]. The Co_3_O_4_-g-C_3_N_4_-150 mg nanocomposite’s elemental composition has been identified using energy-dispersive X-ray spectroscopy (EDS). The EDS spectra of the Co_3_O_4_-g-C_3_N_4_-150 mg nanocomposite’s nanoparticles are shown in Figure 5e, with strong peaks for carbon, nitrogen, cobalt, and oxygen. These peaks demonstrate the phase creation of the Co_3_O_4_-g-C_3_N_4_-150 mg nanocomposite, and the EDS results agree with the XRD and XPS investigations, indicating the formation of the phase.

### 3.5. Electrochemical Study

**Figure 6:**
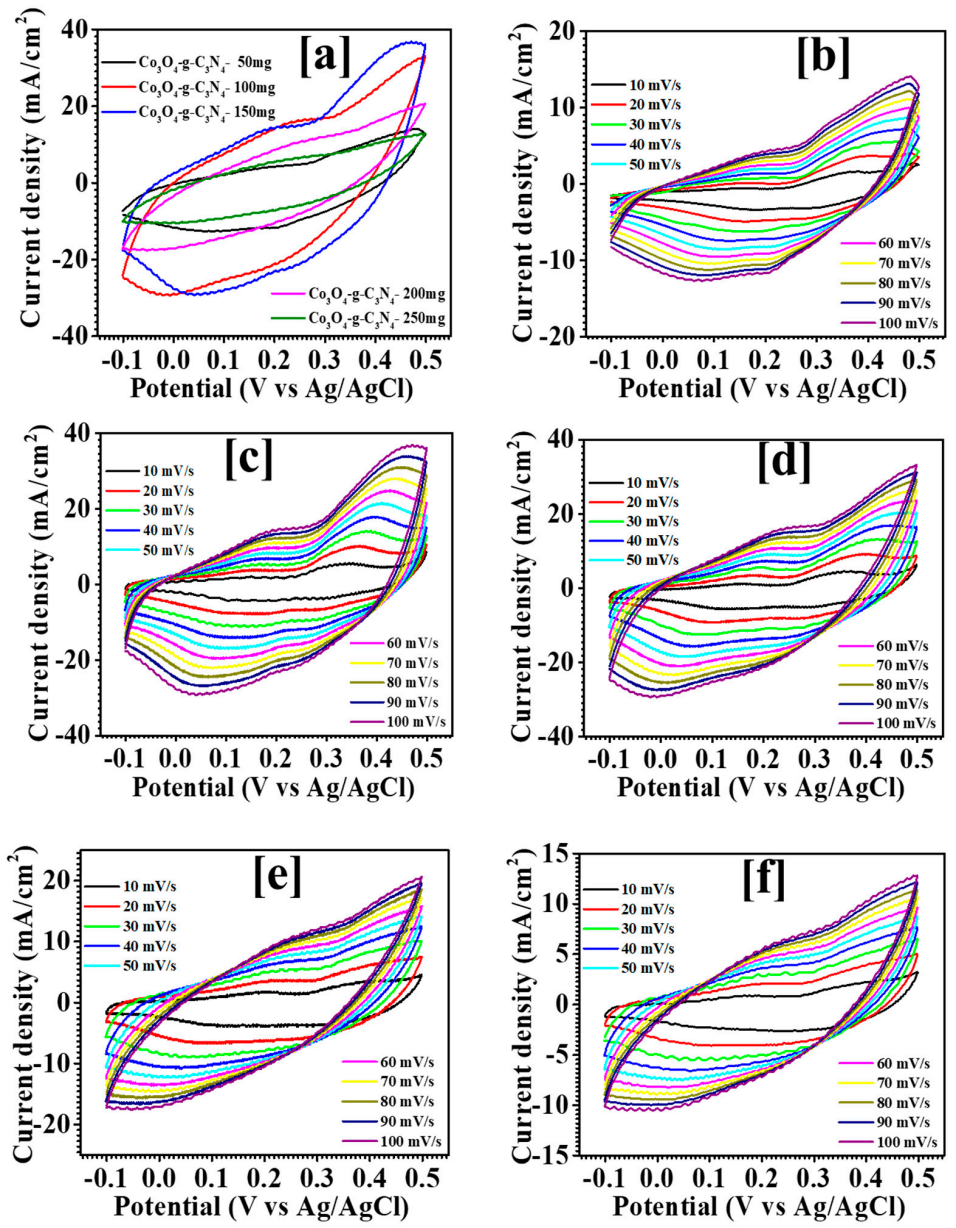
(**a**) Comparative CV profile; (**b**–**f**) CV profile at different scan rates of Co_3_O_4_-g-C_3_N_4_ composite for different weight percentages of g-C_3_N_4_ nanoparticles.

**Figure 7:**
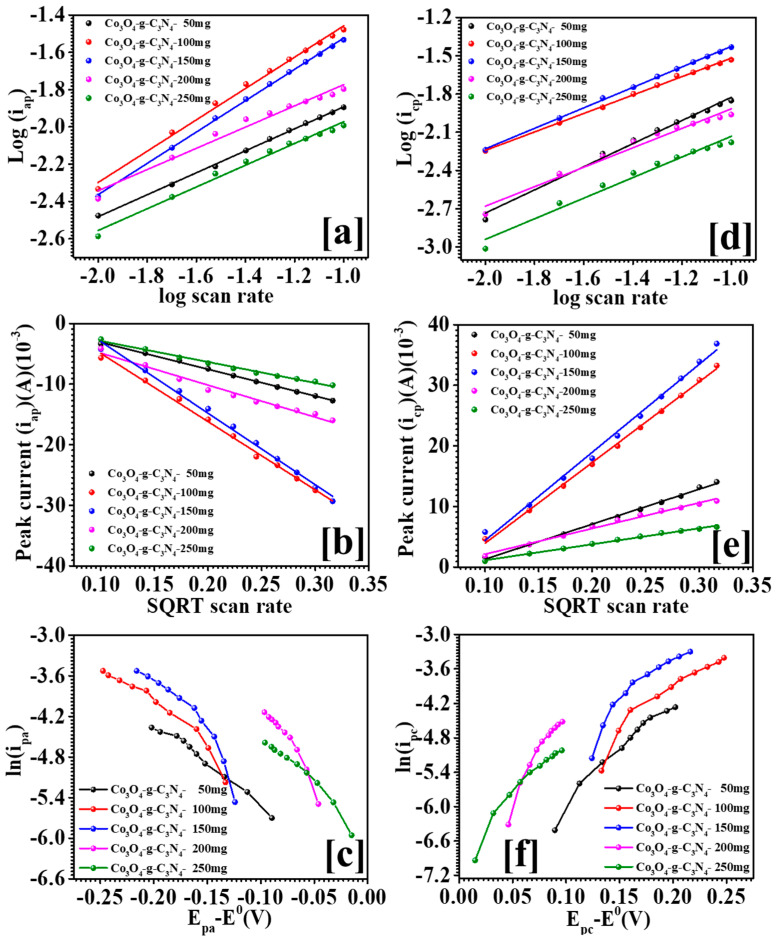
(**a**,**d**) Plot for b value; (**b**,**e**) plot for diffusion coefficient; (**c**,**f**) plot for k^0^ and α of Co_3_O_4_-g-C_3_N_4_ composite for different weight percentages of g-C_3_N_4_ nanoparticles.

In an aqueous potassium hydroxide electrolyte (2 M KOH), and adopted from the literature [34,35], the electrochemical performance of the Co_3_O_4_-g-C_3_N_4_ nanocomposite electrode was examined utilizing cyclic voltammetry (CV), galvanostatic charge–discharge (GCD), and electrochemical impedance spectroscopy (EIS). A SP5 Wonatech workstation was employed to examine the electrochemical performance of all electrodes. Figure 6a–f depicts the comparative and individual cyclic voltammetry (CV) profiles of all electrodes. The redox peaks in all electrode CV profiles represent charge storage via a pseudocapacitor charge storage process. The anode and cathode redox peaks in the CV profiles shift to the negative and positive sides of the formal potential value, respectively. The reversibility of the electrochemical reaction is indicated by an increase in the area under the CV profile as the scan rate increases. Figure 6a additionally illustrates that as the weight percentage of g-C_3_N_4_ in the Co_3_O_4_ nanoparticle composite expands so does the area under the CV profile. Surface morphological changes are readily visible in the FESEM analysis at 50 mg of g-C_3_N_4_. From the FESEM results, the sheets of g-C_3_N_4_ nanoparticles are partially linked with the Co_3_O_4_ nanoparticles, implying that g-C_3_N_4_ nanoparticles operate as a surface for the growth of Co_3_O_4_ nanoparticles. Co_3_O_4_ nanoparticle wires produce a network-like structure, making it easier for electrolytes and electrode materials to interact by removing impediments in the charging and discharging process. The CV profile of the Co_3_O_4_-g-C_3_N_4_ nanocomposite electrode shows this effect. The area of the CV profile increases as the weight percentage of g-C_3_N_4_ in the Co_3_O_4_-g-C_3_N_4_ nanocomposite increases up to 100 mg. The FESEM micrograph shows a huge number of g-C_3_N_4_ particles over a wide surface area, making arranging Co_3_O_4_ nanoparticles easier. Furthermore, some g-C_3_N_4_ particles have a broken floral structure and begin to mix with one another, increasing the total nanoparticle and nanowire area of Co_3_O_4_. This improved electrical network promotes ion intercalation and deintercalation, increasing the size of the CV profile for the Co_3_O_4_-g-C_3_N_4_-100 mg electrode. The structure of the microstructure was partially destroyed as the weight percentage of the g-C_3_N_4_ nanoparticles grew to 150 mg, and nanowires were dispersed across the g-C_3_N_4_ sheets. The increase in the CV profile of the Co_3_O_4_-g-C_3_N_4_-150 mg electrode had some consequences. On the surface of the g-C_3_N_4_ sheet, relatively few flower nanowires of Co_3_O_4_ were visible upon increasing the weight percentage to 200 mg and 250 mg of g-C_3_N_4_ nanoparticles. This was observed to have the impact of lowering the Co_3_O_4_-g-C_3_N_4_ nanocomposite’s area under the CV profile. Furthermore, all electrodes’ kinetics have been investigated by analyzing the CV profile and determining the b value. The b value dictates the charge storage of the electrode material, either a capacitive or a diffusion contribution. A b value of 0.5 represents charge storage by diffusion, whereas 1.0 represents charge storage via a capacitive contribution [36,37,38]. Using the CV profile in Figure 7a,d, the b values for all electrodes were computed from Equation (1).

The b values ranged between 0.58 and 0.84, increasing as the weight percentage of g-C_3_N_4_ nanoparticles grew. The larger b value may be due to the increased surface area of g-C_3_N_4_, which affects the electrode’s capacitive contribution. Table 1 shows the b values for all electrodes. Equation (2) and Figure 7b,e were used to compute the diffusion coefficient of all g-C_3_N_4_ nanoparticles. Table 1 contains a list of all diffusion coefficient values. The Co_3_O_4_-g-C_3_N_4_-150 mg electrode has a diffusion coefficient of 7.8 × 10^−8^. 

The electrochemical reversibility of all electrodes was also investigated using the CV profile. With respect to the standard rate constant (k^0^), the chemical processes are defined as reversible, irreversible, or quasi-reversible. k^0^ is between 10^−1^ and 10^−5^ for quasi-reversible processes, less than 10^−1^ for reversible reactions, and larger than 10^−5^ for irreversible reactions [39,40]. Using the CV profile, the k^0^ values were computed from Equation (3) and Figure 7c,f, and all electrodes revealed k^0^ values in the 10^–5^ range, confirming electrochemical charge storage through a quasi-reversible reaction mechanism. The k^0^ values fell from 0.75 × 10^−5^ to 1.78 × 10^−5^ as the weight percentage of g-C_3_N_4_ in the composite increased. This might be related to the alteration of the surface microstructure of the Co_3_O_4_-g-C_3_N_4_ nanocomposite by altering the g-C_3_N_4_ weight percentage. In order to comprehend the reaction process, the transfer coefficient of the electrodes was determined as well. The transfer coefficient was computed using Equation (3) [39] and, although it ranged between 0.0 and 1.0, all electrodes displayed quasi-reversible response mechanisms. Table 1 displays the transfer coefficient values. 

**Figure 8:**
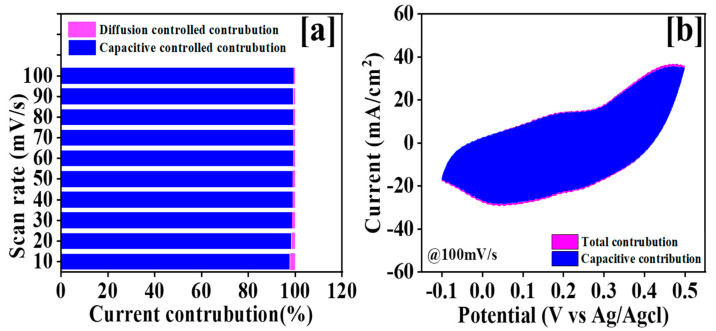
(**a**) Diffusion and capacitive-controlled contributions at different scan rates and (**b**) total and capacitive-controlled contribution at 100 mV/s scan rate for the Co_3_O_4_-g-C_3_N_4_-150 mg electrode.

The optimized electrode’s b value shows that the surface capacitive contribution to charge accumulation was dominant. Equation (4) [39] was used to compute the capacitive and diffusion contributions of the current at constant potential. At a scan rate of 10 mV/s, the capacitive contribution is 97.92%, which corresponds to the electrode’s b value. Figure 8a shows the capacitive and diffusive-controlled contributions at various scan rates, while Figure 8b shows the total and capacitive-controlled contributions of the CV profile at 100 mV/s. 

**Figure 9:**
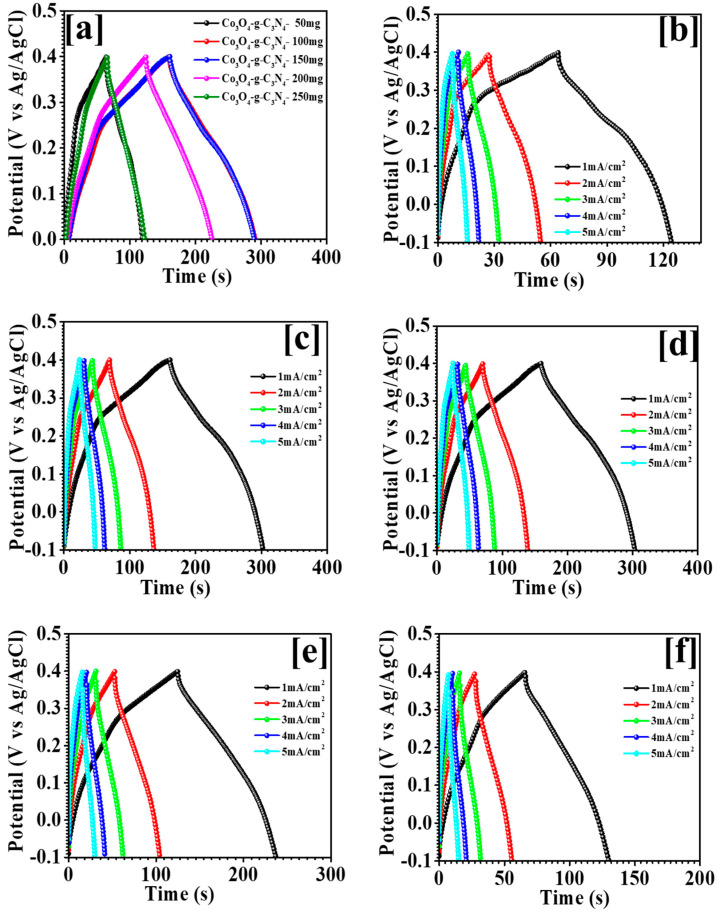
(**a**) Comparative GCD profile; (**b**–**f**) GCD profile at different current density of Co_3_O_4_-g-C_3_N_4_ composite for different weight percentages of g-C_3_N_4_ nanoparticles.

The specific capacitance (C_s_), energy density (ED_s_), and power density (PD_s_) of the electrode materials have been determined from the GCD profiles of all electrodes. Figure 9a–e illustrates the GCD curves of the Co_3_O_4_-g-C_3_N_4_ nanocomposite electrode and individual electrodes in comparison. Comparing the GCD profile, the Co_3_O_4_-g-C_3_N_4_ nanocomposite electrode, synthesized with a 150 mg weight of g-C_3_N_4_, has a higher time to discharge than the other electrodes. Regarding the charging and discharging times of the electrode, the influence of morphological alteration by tweaking the weight percentage of g-C_3_N_4_ was observed.

**Figure 10:**
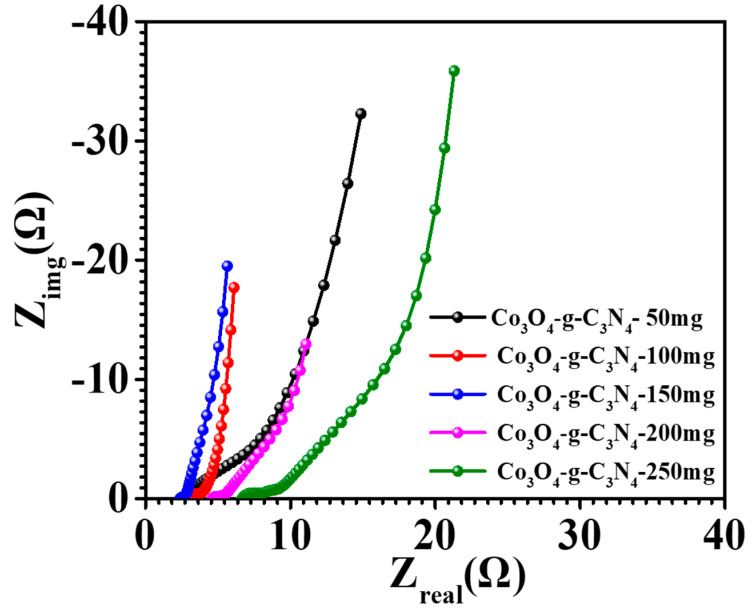
EIS spectra of Co_3_O_4_-g-C_3_N_4_ composite for different weight percentages of g-C_3_N_4_ nanoparticles.

The electrode with a 50-mg weight percentage of g-C_3_N_4_ has shorter charging and discharging periods, and the electrode with a 150-mg weight percentage of g-C_3_N_4_ has a longer discharge time, probably owing to the electrode’s improved surface modification of Co_3_O_4_ nanoparticles. Using Equations (5)–(7) [41,42,43,44], the specific capacitance, energy density, and power density of all electrodes were computed and are listed in Table 2. At a current density of 1 mA/cm^2^, the Co_3_O_4_-g-C_3_N_4_ nanocomposite electrode with 150 mg g-C_3_N_4_ has a specific capacitance of 198 F/g, which drops as it grows in current density. Figure 10 depicts the electrochemical impedance spectroscopy (EIS) of all Co_3_O_4_-g-C_3_N_4_ nanocomposite electrodes. The EIS spectra are split into three sections: series resistance (R_s_), charge transfer resistance (CTR), and Warburg. The intercept on the x-axis was used to calculate the R_s_ value. Table 2 shows the R_s_ values for all electrodes. The R_s_ value of the Co_3_O_4_-g-C_3_N_4_-150 mg electrode is lower, presumably owing to the greater sheet area accessible for the electrode supplied by the g-C_3_N_4_ sheet and the dispersion of nanowires over the nanosheets.

### 3.6. Electrochemical Study of an Asymmetric Supercapacitor

By fabricating an asymmetric supercapacitor (ASC) with the optimized Co_3_O_4_-g-C_3_N_4_ nanocomposite electrode as the working electrode and activated carbon material as the counter electrode, the practical application of the optimal Co_3_O_4_-g-C_3_N_4_-150 mg nanocomposite electrode was examined.

**Figure 11:**
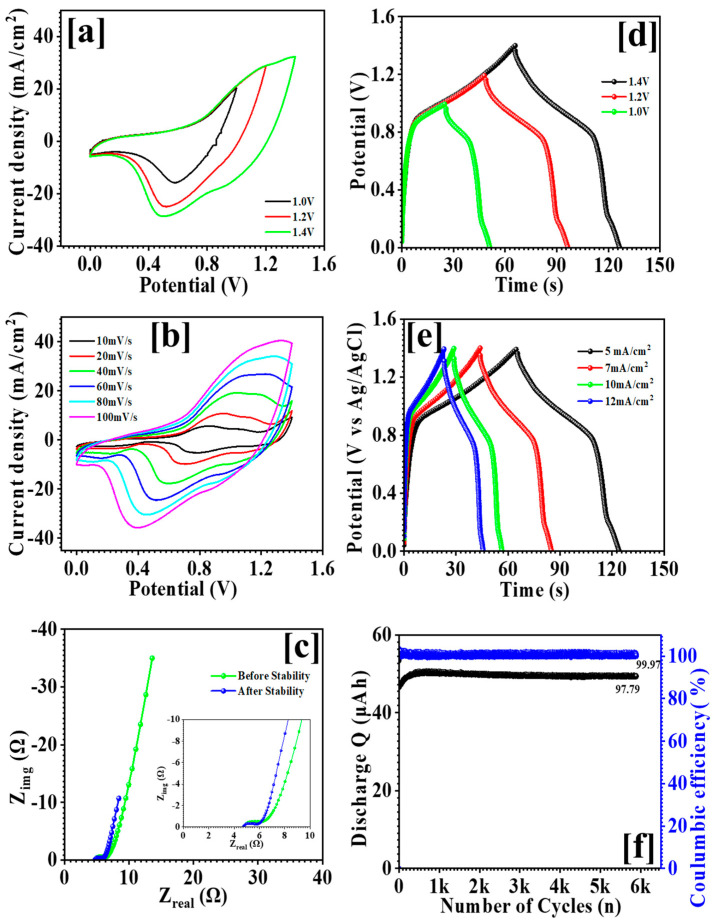
(**a**) CV profile at different potential windows; (**b**) CV profile at different scan rates; (**c**) EIS spectra before and after stability; (**d**) GCD profile at different potential windows; (**e**) GCD profile at different current densities; (**f**) cyclic stability over 6K GCD cycles of an asymmetric supercapacitor device.

The thorough electrode preparation process for activated carbon is quite similar to that for active electrode material; however, activated carbon was used in the AC electrode preparation instead of active material and referred to as Co_3_O_4_-g-C_3_N_4_-150 mg//Activated carbon (ASC). The ASC was studied using the prepared devices. Figure 11a illustrates the cyclic voltammetry (CV) profile with different potential ranges ranging from 1.0 volt to 1.4 volts, and the final potential window was taken from Figure 11a and kept at 1.4 volts. A similar approach was used to calculate the galvanostatic charge–discharge (GCD) profile at various potentials, as shown in Figure 11d. All ASC supercapacitor electrochemical investigations were carried out with the potential window set to 1.4 volts. Figure 11b depicts the CV profile at a 1.4 V potential window with different scan rates ranging from 10 mV/s to 100 mV/s. Figure 11e shows the ASC’s GCD curve at various current densities. Using the ASC’s GCD profile, the specific capacitance, energy density, and power density were determined using the equations. At a current density of 5 mA/cm^2^, the ASC’s specific capacitance is 43 F/g. At a power density of 701 W/kg, the ASC has an energy density of 11.86 Wh/kg. Figure 11c depicts the ASC electrochemical impedance spectroscopy (EIS) spectrum. The image demonstrates the EIS spectra of the ASC before and after 6k cycles of stability testing. The ASC’s R_s_ value before stabilization is 4.7 Ωcm^2^, and it rises to 4.46 Ωcm^2^ after stabilization. The minor rise in the R_s_ value after stabilization might be attributable to the surface alteration of the nanoparticles. Figure 11f depicts the ASC’s cyclic stability and columbic efficiency over 6k cycles. The cyclic stability is 97.79%, and the ASC’s columbic efficiency is 99.97%. Overall, the electrochemical investigation and evaluation of the Co_3_O_4_-g-C_3_N_4_-150 mg nanocomposite electrode for three-electrode and two-electrode configurations show that the Co_3_O_4_-g-C_3_N_4_-150 mg nanocomposite electrode is a viable electrode material in energy storage applications.

## 4. Conclusions

The hydrothermal method was used to effectively prepare a Co_3_O_4_-g-C_3_N_4_-150 mg composite electrode in the present study. The Co_3_O_4_-g-C_3_N_4_ composite electrode with a weight proportion of g-C_3_N_4_ of 150 mg had a good specific capacitance of 198 F/g. The Co_3_O_4_-g-C_3_N_4_-150 mg electrode, an activated carbon electrode, and a polyvinyl alcohol with potassium hydroxide gel were used to construct an asymmetric supercapacitor. The asymmetric supercapacitor’s specific capacitance was measured at 43 F/g, with a remarkable energy density of 11.86 Wh/kg and a power density of 701 W/kg. The asymmetric supercapacitor’s galvanostatic charge–discharge (GCD) cyclic stability demonstrated excellent capacity retention of 97.79% over 6000 cycles and a columbic efficiency of 99.97%. The remarkable efficiency of the asymmetric supercapacitor constructed with the Co_3_O_4_-g-C_3_N_4_-150 mg electrode indicates that Co_3_O_4_-g-C_3_N_4_ composite electrodes are beneficial for energy storage applications.

## Figures and Tables

**Table 1:** Comparative b value, transfer coefficient (α), and diffusion coefficient (D) of all Co_3_O_4_-g-C_3_N_4_ composite electrode.

Sample Code	b Value	Transfer Coefficient (α)	Diffusion Coefficient (D) (cm/S) × 10^−8^
Co_3_O_4_-g-C_3_N_4_-50 mg	0.58	0.31	1.16
Co_3_O_4_-g-C_3_N_4_-100 mg	0.72	0.33	6.16
Co_3_O_4_-g-C_3_N_4_-150 mg	0.84	0.48	7.37
Co_3_O_4_-g-C_3_N_4_-200 mg	0.56	0.46	0.55
Co_3_O_4_-g-C_3_N_4_-250 mg	0.80	0.40	0.23

**Table 2:** Comparative specific capacitance (C_s_), energy density (ED), power density (PD) and equivalent series resistance (ESR) of all Co_3_O_4_-g-C_3_N_4_ composite electrodes.

Sample Code	Specific Capacitance (C_s_) F/g	Energy Density (ED) Wh/kg	Power Density (PD) W/g	ESR (R_s_) (Ωcm^2^)
Co_3_O_4_-g-C_3_N_4_-50 mg	93	3.25	192	2.86
Co_3_O_4_-g-C_3_N_4_-100 mg	189	6.57	166	2.82
Co_3_O_4_-g-C_3_N_4_-150 mg	198	6.85	166	2.34
Co_3_O_4_-g-C_3_N_4_-200 mg	152	5.27	166	4.54
Co_3_O_4_-g-C_3_N_4_-250 mg	86	3.01	166	6.59

## Data Availability

All the relevant data are included in this published article.
